# Dissociable Behavioral, Physiological and Neural Effects of Acute Glucose and Fructose Ingestion: A Pilot Study

**DOI:** 10.1371/journal.pone.0130280

**Published:** 2015-06-24

**Authors:** Bettina Karin Wölnerhanssen, Anne Christin Meyer-Gerspach, André Schmidt, Nina Zimak, Ralph Peterli, Christoph Beglinger, Stefan Borgwardt

**Affiliations:** 1 Department of Gastroenterology, University Hospital of Basel, Basel, Switzerland; 2 Medical Image Analysis Center, University Hospital of Basel, Basel, Switzerland; 3 Department of Psychiatry, University Hospital of Basel, Basel, Switzerland; 4 Department of Surgery, St. Clara Hospital, Basel, Switzerland; Vanderbilt University, UNITED STATES

## Abstract

Previous research has revealed that glucose and fructose ingestion differentially modulate release of satiation hormones. Recent studies have begun to elucidate brain-gut interactions with neuroimaging approaches such as magnetic resonance imaging (MRI), but the neural mechanism underlying different behavioral and physiological effects of glucose and fructose are unclear. In this paper, we have used resting state functional MRI to explore whether acute glucose and fructose ingestion also induced dissociable effects in the neural system. Using a cross-over, double-blind, placebo-controlled design, we compared resting state functional connectivity (rsFC) strengths within the basal ganglia/limbic network in 12 healthy lean males. Each subject was administered fructose, glucose and placebo on three separate occasions. Subsequent correlation analysis was used to examine relations between rsFC findings and plasma concentrations of satiation hormones and subjective feelings of appetite. Glucose ingestion induced significantly greater elevations in plasma glucose, insulin, GLP-1 and GIP, while feelings of fullness increased and prospective food consumption decreased relative to fructose. Furthermore, glucose increased rsFC of the left caudatus and putamen, precuneus and lingual gyrus more than fructose, whereas within the basal ganglia/limbic network, fructose increased rsFC of the left amygdala, left hippocampus, right parahippocampus, orbitofrontal cortex and precentral gyrus more than glucose. Moreover, compared to fructose, the increased rsFC after glucose positively correlated with the glucose-induced increase in insulin. Our findings suggest that glucose and fructose induce dissociable effects on rsFC within the basal ganglia/limbic network, which are probably mediated by different insulin levels. A larger study would be recommended in order to confirm these findings.

## Introduction

Functional MRI is a rather novel method to assess brain activity after oral intake of defined nutrients to examine physiological gut-brain interactions [1,2]. Appetite regulation is mediated via a functional interplay between homeostatic and non-homeostatic brain areas [3,4]. Besides the hypothalamus as the central gateway, reward-related brain regions such as the striatum or the orbitofrontal cortex (OFC) have also been implicated in feeding behavior [5,6]. In particular, it has been suggested that striatal responses to food may reflect the hedonic and rewarding value of feeding, while other regions including the OFC, amygdala and hippocampus may be more related to motivational and cognitive aspects of food control [7,8]. Responses in these brain regions depend on levels of peripheral satiation hormones [9,10]. In order to maintain appropriate levels of energy balance, ingested nutrients trigger a variety of satiation signals (e.g. GIP, gastric inhibitory polypeptide; GLP-1, glucagon-like peptide-1) with immediate effects on appetite, whereas adiposity signals (e.g. leptin and insulin) are responsible for the long-run maintenance of energy balance [4,11]. Recent studies show that insulin and leptin reduce reward-driven food intake as well and thus also have an immediate appetite-suppressing effect [12,13].

Fructose is a monosaccharide naturally found in honey and fruits. High-fructose corn syrup—a mixture of glucose and fructose in varying concentrations—is increasingly used in soft drinks and is partially held responsible for the worldwide increase in fructose consumption. Chronic fructose consumption may adversely affect human health by leading to increased de novo lipogenesis in the liver, hyperuricemia, insulin resistance and obesity [14,15]. Fructose consumption might even contribute to continuous food intake and exert symptoms of tolerance and withdrawal by down regulation of dopamine receptors in reward-sensitive pathways [16,17]. Glucose is a highly potent secretagogue leading to release of insulin and satiation peptides by enteroendocrine cells and inhibits the release of the appetite inducer ghrelin. In contrast, fructose intake does not affect the release of the above-mentioned peptides to the same extent [18,19] and rather than suppressing the intake of additional food, calories from fructose seem to add on to the total calorie intake [20].

The global obesity problem supports the urgent need for research that aims to understand the basic mechanisms that regulate food intake, appetite and body weight. However, it is unclear how different behavioural and physiological responses to glucose and fructose are mirrored in the neural system including sensory, cognitive and reward processes. Therefore, we are exploring the role of ingested nutrients in triggering adaptive processes in the brain by uncovering the temporal relations between gut and brain signals that control eating and feeding behaviour and energy consumption.

To address this question, we used resting state functional MRI to examine neural changes after the acute ingestion of fructose in comparison with glucose. Resting state functional connectivity (rsFC) is based on the analysis of low-frequency fluctuations present in the blood-oxygenation-level-dependent signal [21]. These low-frequency fluctuations have been shown to be temporally correlated within spatially distinct but functionally related resting state networks establishing an intrinsic functional architecture [22]. Resting state functional connectivity analysis is particularly suitable to examine brain functions including sensory, cognitive and reward processes [23,24]. Previous resting state fMRI studies have detected a basal ganglia/limbic network during rest, which subsumes the striatum, the thalamus and the amygdala [25,26]. Many of these regions are strongly implicated in reward processes and dopamine function [27].

Primary outcome of this study was to examine differences between glucose and fructose administration with respect to resting state functional connectivity within the basal ganglia/limbic network. The secondary outcome was to examine whether these effects are related to the behavioral and physiological effects of glucose and fructose. We hypothesized that fMRI would show a different pattern of activation within the neural reward network after fructose versus glucose ingestion.

## Materials and Methods

The original protocol is included under supplemental material. Morbidly obese volunteers have also been examined; however, this data is not included in this manuscript as it is currently being analyzed.

### Subjects

Twelve right-handed male volunteers (mean age: 24.8 years, range: 21–31 years and mean BMI: 22.9 kg/m^2^, range: 21–24.0 kg/m^2^) were analyzed. The protocol was approved by the Ethics Committee of Basel, Switzerland (EKBB: 08/11) and conducted in accordance with the principles of the Declaration of Helsinki. Subjects were recruited by word of mouth over a period of three months (March 2012- June 2012). Each participant underwent a medical interview, laboratory screening and gave written informed consent. Exclusion criteria were: smoking, substance abuse, regular intake of medications, medical or psychiatric illness, fructose intolerance and any contraindication to MRI (e.g. claustrophobia, non-removable metal devices), and any abnormalities detected upon laboratory screening. Of the fourteen subjects originally recruited two had to be excluded as they did not meet the eligibility criteria. There was also one drop-out who was replaced. Hence, complete data from 12 subjects were obtained for analysis. The purpose of this study is to gain basic information on the physiologic role of the aforementioned carbohydrates on regional blood flow in the brain. Samples size of this study was chosen on the basis of practical considerations rather than statistical estimation. However, according to our experience, a sample size of 12 subjects will most likely allow to detect large differences in parameters (> 50%) between the treatments groups.

### Study design and experimental procedures

The study was a randomized, placebo-controlled, double-blind, cross-over trial and was carried out at the Phase one Research Unit of the University Hospital of Basel. Each subject was administered fructose, glucose and placebo on three separate occasions. The treatment order was randomized within a subject and the time between these visits was at least 7 days.

After an overnight fast of at least 10 hours, an 8F polyvinyl nasogastric tube was inserted into the stomach through an anaesthetized nostril and its intragastric position was confirmed by rapid injection of 10ml of air and auscultation of the upper abdomen. A peripheral venous cannula was inserted for blood sample collection. Two baseline blood samples were taken and appetite perceptions (feelings of: a) hunger, b) satiety, c) fullness and d) prospective food consumption) were assessed by visual analogue scales (VAS) [28]. The solutions were freshly prepared and were at room temperature when administered. Glucose monohydrate and fructose were purchased from Haenseler AG (Herisau, Switzerland). In order to maintain the blind differing persons prepared and administered the treatment. Subjects received 300ml of tap water with 75g of glucose or with 25g of fructose, or 300ml pure tap water (placebo) via nasogastric tube over 2 minutes while sitting in the MR room. After administration, the feeding tube was removed and the subjects underwent resting state MRI scanning, which began within 5 minutes after administration of the test solution. After 15 and 60 minutes blood samples were taken and VAS recorded. Visual analogue scales consisted of a horizontal, unstructured, 10-cm line representing the minimum (0.0 points) to the maximum rating (10.0 points). Subjects assigned a vertical mark across the line to indicate the magnitude of their subjective sensation at the present time point. The measurement was quantified by the distance from the left end of the line (minimum rating) to the subject’s vertical mark.

Blood samples were taken after glucose and fructose treatment, no blood samples were collected after placebo treatment. Subjects were told that blood sampling was randomized and hence did not associate no blood samples with placebo treatment. Blood samples were collected into chilled tubes containing EDTA (6 μmol/L blood), a protease-inhibitor cocktail (Complete, EDTA-free, 1 tablet/50mL blood, Roche, Mannheim, Germany), and a dipeptidyl peptidase IV inhibitor (10μl/mL blood, Millipore Corp., St. Charles, Missouri, U.S.A). After centrifugation (3000rpm, 10 min at 4 C), plasma samples were processed into different aliquots and kept frozen at -70 C until analysis. Blood pressure and heart rate were measured before and after each study day.

### Laboratory analysis

Active GLP-1 was measured with a commercially available ELISA kit (Millipore, Billerica, MA). This kit is for non-radioactive quantification of GLP-1 (7–36) in serum and EDTA plasma samples; it is highly specific and does not detect other forms of GLP-1. The lowest level of GLP-1 that can be detected by this assay is 0.5 pmol/L when a 100-μL plasma sample is used. Insulin was measured with a commercially available radioimmunoassay kit (CIS Bio International, Bagnols, France). This kit is for quantitative determination of insulin in human serum and plasma (EDTA), is highly specific for insulin and shows no cross-reactivity with other peptides, e.g., C-peptide or glucagon. The intra- and interassay coefficients of variation for this assay are <12.2 and 9.0%, respectively. The lowest level of insulin that can be detected by this assay is 4.6 μU/mL. Plasma glucose concentration was measured by a commercially available glucose oxidase method (Bayer Consumer Care, Basel, Switzerland). This method is highly specific for measurement of glucose in serum or plasma. The lowest level of glucose that can be detected by this assay is 0.6 mmol/L. GIP was measured with a commercially available ELISA kit (Millipore, Billerica, MA, U.S.A.). This kit is used for non-radioactive quantification of human GIP in serum and EDTA plasma samples and has 100% cross reactivity to human GIP (1–42) and GIP (3–42). The lowest level of GIP that can be detected by this assay is 4.2 pg/mL when a 20-μL plasma sample is used. The intra and inter-assay coefficients of variation for this assay are below 8.8 and 6.1%, respectively.

### Resting state functional MRI

For the resting-state scan (5 minutes), subjects were instructed to lie in dimmed light with their eyes open, to think of nothing in particular, and not to fall asleep.

### Image acquisition

Scanning was performed on a 3 T scanner (Siemens Magnetom Verio) 5 minutes post-treatment. Whole-brain functional imaging was performed using a gradient echo planar imaging (EPI) sequence (TR = 2000 ms, TE = 28 ms, flip angle = 82°, field of view = 228 x 228 mm^2^, 32 slices, slice thickness: 3.3 mm; voxel size = 3.6 x 3.6 x 3.3 mm^3^). In total, 152 EPI volumes were acquired. Additionally, a high-resolution T1-weighted magnetization prepared rapid acquisition gradient echo (MPRAGE) image was acquired (TR = 2000 ms; TE = 3.37 ms; flip angle = 8°; inversion time = 1000 ms; 176 slices; slice thickness = 1 mm; voxel size = 1 x 1 x 1 mm^3^).

### Functional connectivity analysis

In a first step, resting state analysis was carried out using MELODIC (Multivariate Exploratory Linear Optimized Decomposition into Independent Components) [23], a part of FSL (FMRIB Software Library, www.fmrib.ox.ac.uk/fsl). MELODIC is a powerful data-driven model-free approach for finding independent patterns in multivariate data (i.e. resting state networks such as the basal ganglia/limbic network). Preprocessing consisted of motion correction using MCFLIRT (Motion Correction using FMRIB's Linear Image Registration Tool) [29], removal of non-brain tissue with BET (Brain Extraction Tool) [30], spatial smoothing using a 5 mm full-width-at-half-maximum Gaussian kernel, and high-pass temporal filtering equivalent to 111.1 seconds. FLIRT was used to register the functional EPI volumes in each individual subject’s high-resolution MPRAGE image and to assign the MPRAGE data to the standard space template (Montreal Neurological Institute, MNI152 T1 1 mm3). Preprocessed functional data containing 152 time points for each subject were temporally concatenated across subjects in order to create a single 4D data set. The data set was decomposed into independent components, with a free estimation for the number of components. The number of subjects in each group was equal, in order to avoid skewing independent components in favor of a particular population/treatment. Group independent component analysis (GICA) was used to identify the basal ganglia/limbic network. This network has been previously noted in other resting state fMRI studies using independent component analysis [25,26]. A dual regression approach [31] with nonparametric permutation (5000) tests (randomize [32], FSL) were carried out to detect statistically significant differences between treatments (placebo vs. fructose, placebo vs glucose, glucose vs fructose) within the boundaries of the spatial map (basal ganglia/limbic network) obtained with GICA. As this is a pilot study with a rather small sample of 12 subjects, we considered a significance threshold of p< 0.05 uncorrected using threshold-free cluster enhancement (TFCE) [33].

### Statistical Analysis

Descriptive statistics were used for demographic variables (age, weight, height, and BMI). Hormone and glucose profiles were analyzed by calculating pharmacodynamic parameters: area under the concentration-time curve (AUC) and ΔAUC. Area under the curve (AUC) was calculated using the trapezoidal method [34]. The parameters were tested for normality by the Shapiro-Wilk test. Hormone profiles (AUC 0–60 min) were compared using Student’s paired t-test. Pearson correlation was used to assess the relation between hormone release 0–15 minutes post-treatment and rsFC. The FC strengths were indexed by parameter estimates (z-scaled) but only of the significant voxels obtained from the treatment comparisons of interest. SPSS for Windows Version 19.0 (SPSS, Chicago, IL) was used for all statistical analysis. Values were reported as means ± SEM; p≤ 0.05 was considered as statistically significant.

## Results

**Fig 1:**
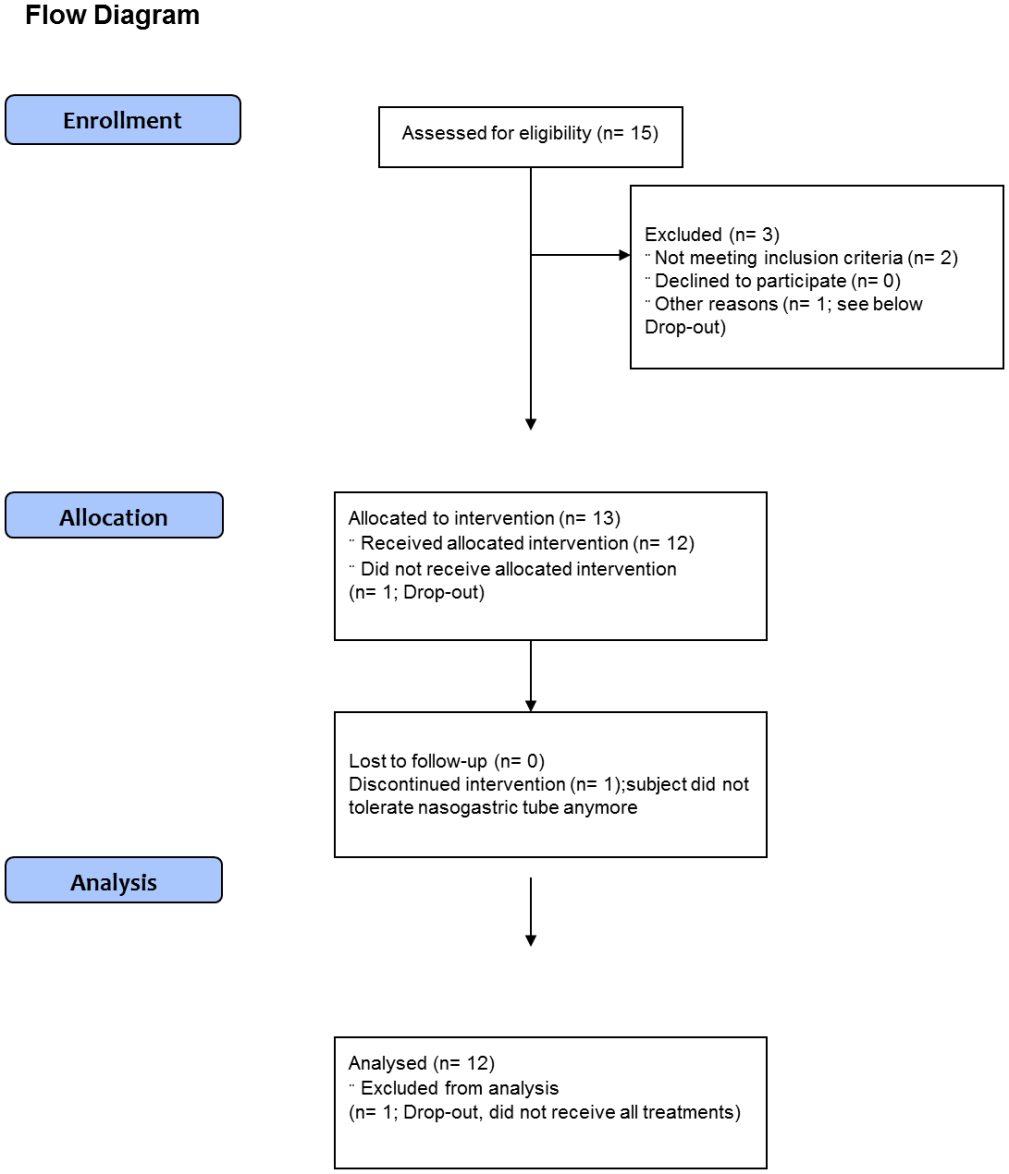
Flowchart. Adapted CONSORT flowchart for clinical trials.

There was one drop-out (one subject completed two study sessions, after which they did not tolerate the nasogastric tube anymore). The data of this person was excluded from analysis and replaced. Complete data from 12 subjects were obtained for analysis. There were no adverse events (Fig 1).

### Behavioral effects

Baseline assessments were not equivalent across all study sessions. Therefore, we used relative values (post-treatment values minus pre-treatment value) representing changes in appetite perception. However, there was no statistically significant difference between baseline values among the three treatment groups.

**Fig 2:**
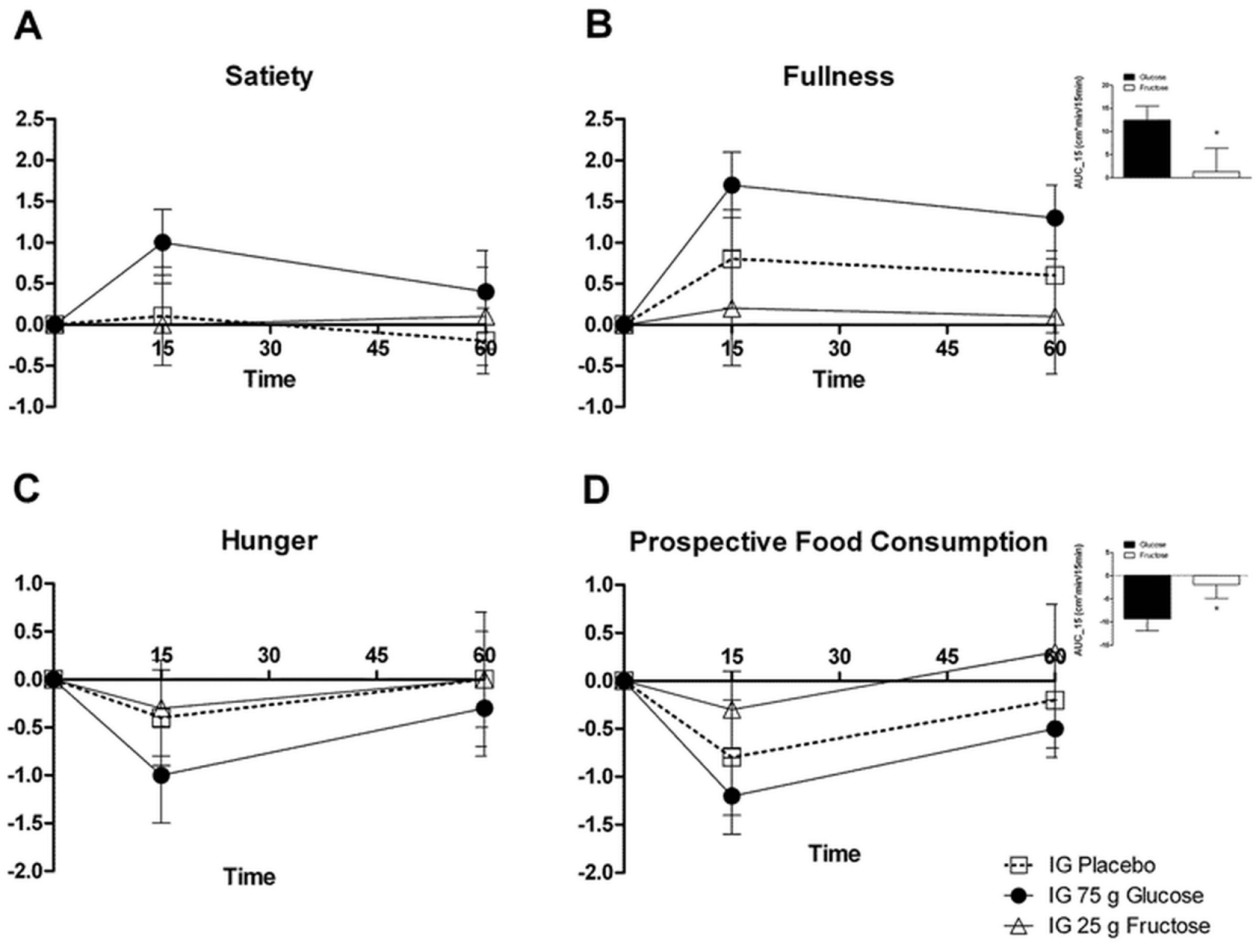
Subjective Appetite Perceptions. Relative to fructose treatments, ingested glucose increased subjective feelings of (A) satiety (n.s.) and (B) fullness (AUC-15 min: p = 0.04) and reduced feelings of (C) hunger (n.s.) and (D) prospective food consumption (AUC-15 min: p = 0.017). Differences seen between placebo and fructose, resp. placebo and glucose were non-significant.

Relative to fructose and placebo, ingested glucose increased feelings of satiety and fullness and reduced feelings of hunger and prospective food consumption. However, these differences were not all statistically significant: fullness was significantly higher after glucose treatment compared to fructose treatment (AUC 0–15 min: p = 0.04) and prospective food consumption was significantly lower after glucose compared to fructose treatment (AUC 0–15 min: p = 0.017). Although feelings of satiety were higher and feelings of hunger were lower after glucose treatment compared to fructose treatment statistical significance was not reached. Interestingly, values after placebo treatment lie in between fructose and glucose. However, differences seen between placebo and fructose, resp. placebo and glucose were non-significant (Fig 2).

### Physiological effects

**Fig 3:**
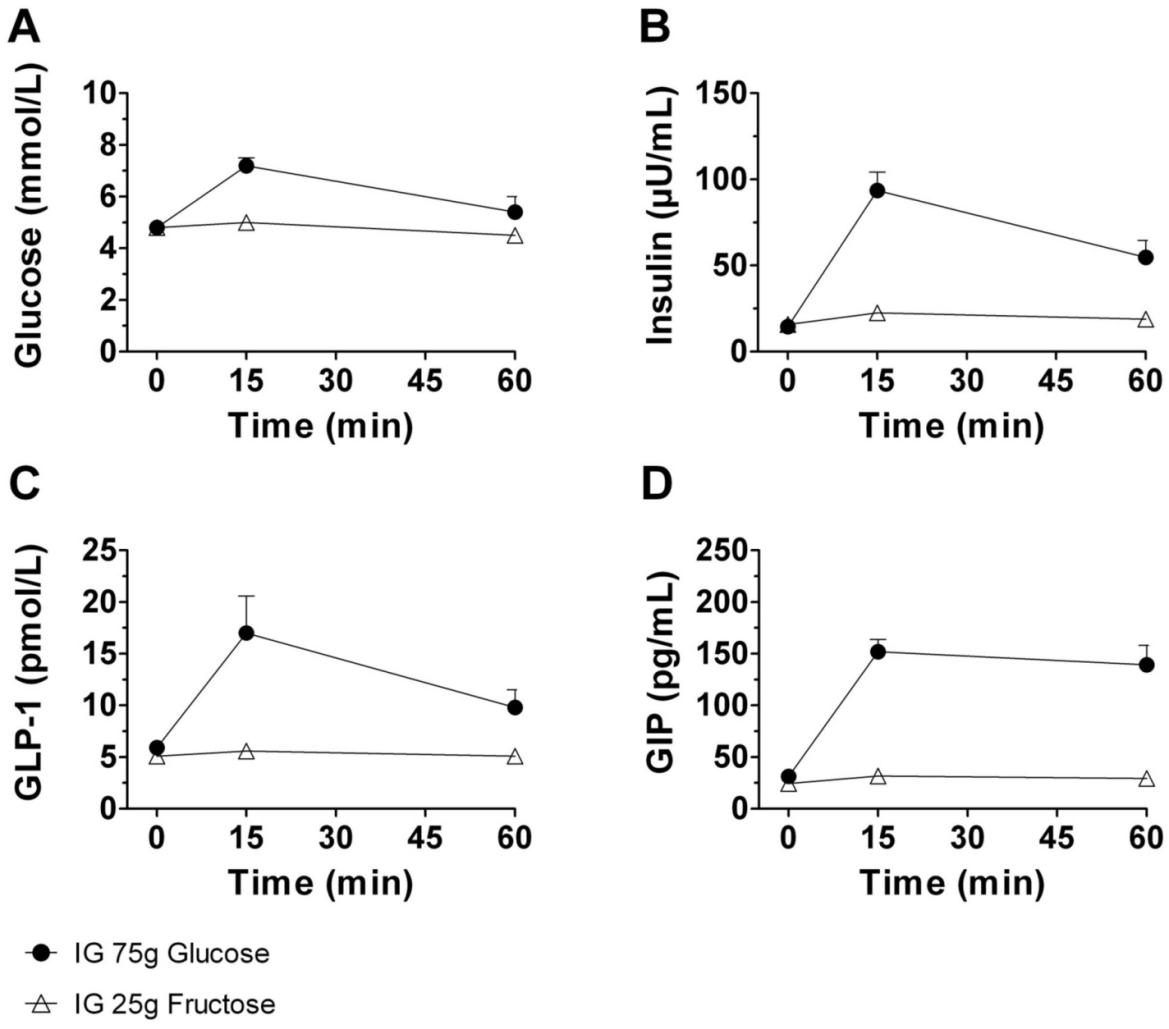
Plasma concentrations of Glucose, Insulin, GLP-1 and GIP after glucose and fructose treatment. (A) Glucose, (B) Insulin, (C) GLP-1 and (D) GIP after oral intake of 75g glucose resp. 25g fructose. Glucose ingestion caused significantly greater elevations in plasma glucose (p = 0.001), insulin (p< 0.001), GLP-1 (p = 0.007), GIP (p< 0.001) concentrations compared to fructose ingestion (AUC 0-60min). Data are expressed as mean ± SEM.

Baseline fasting values of plasma glucose, insulin, GLP-1 and GIP were not significantly different across all study sessions (compared using repeated-measures analysis of variance ANOVA with Bonferroni correction). Glucose ingestion caused significantly higher elevations of plasma glucose (p = 0.001), insulin (p< 0.001), GLP-1 (p = 0.007) and GIP (p< 0.001) concentrations compared to fructose ingestion (AUC 0–60 min; Fig 3).

### Neural effects

**Fig 4:**
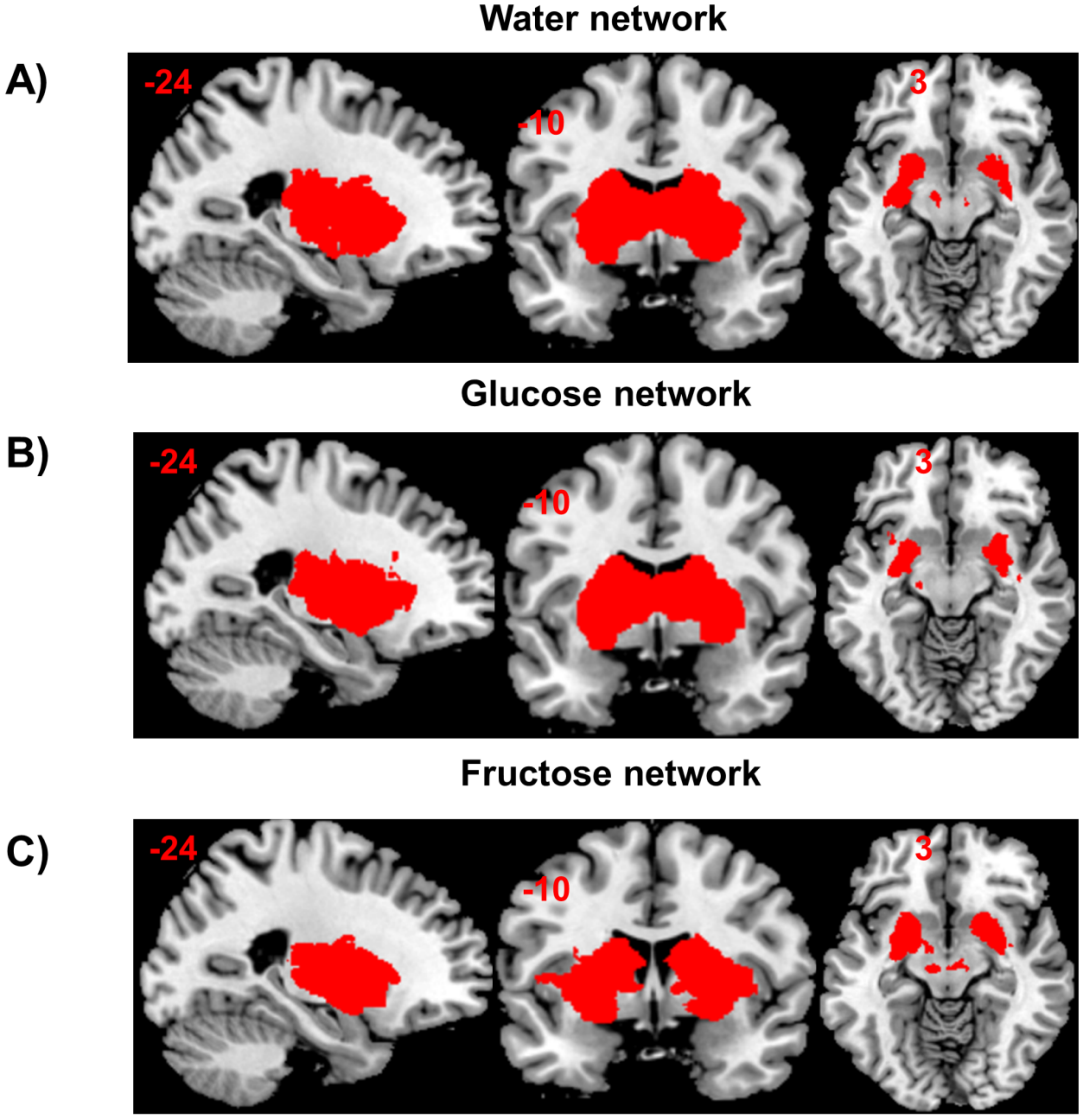
Spatial maps representing the resting state basal ganglia/limbic network for each treatment condition. Spatial maps representing the reward network detected by GICA for (A) the placebo (i.e. water), (B) glucose and (C) fructose treatment. Maps were created using a one-sample t-test for each treatment (randomize, FWE-corrected at p = 0.001). Regions belonging to this network include the entire striatum, thalamus, and amygdala. The right side of the brain is displayed on the right side of the figure.

After glucose ingestion, dual regression of the basal ganglia/limbic network identified with GICA (Fig 4) revealed increased rsFC of the right caudatus, left pallidum and OFC to this network, relative to placebo. After placebo increased rsFC of the angular gyrus, lateral occipital cortex and precuneus was found to the basal ganglia/limbic network, relative to glucose. After fructose, increased rsFC of the OFC, cerebellum and lateral occipital cortex was found, relative to placebo. After placebo, increased rsFC of the superior parietal lobule, paracingulate gyrus and inferior frontal cortex to the basal ganglia/limbic network was found relative to fructose. Treatment comparisons relative to placebo are summarized in S1 Table.

**Fig 5:**
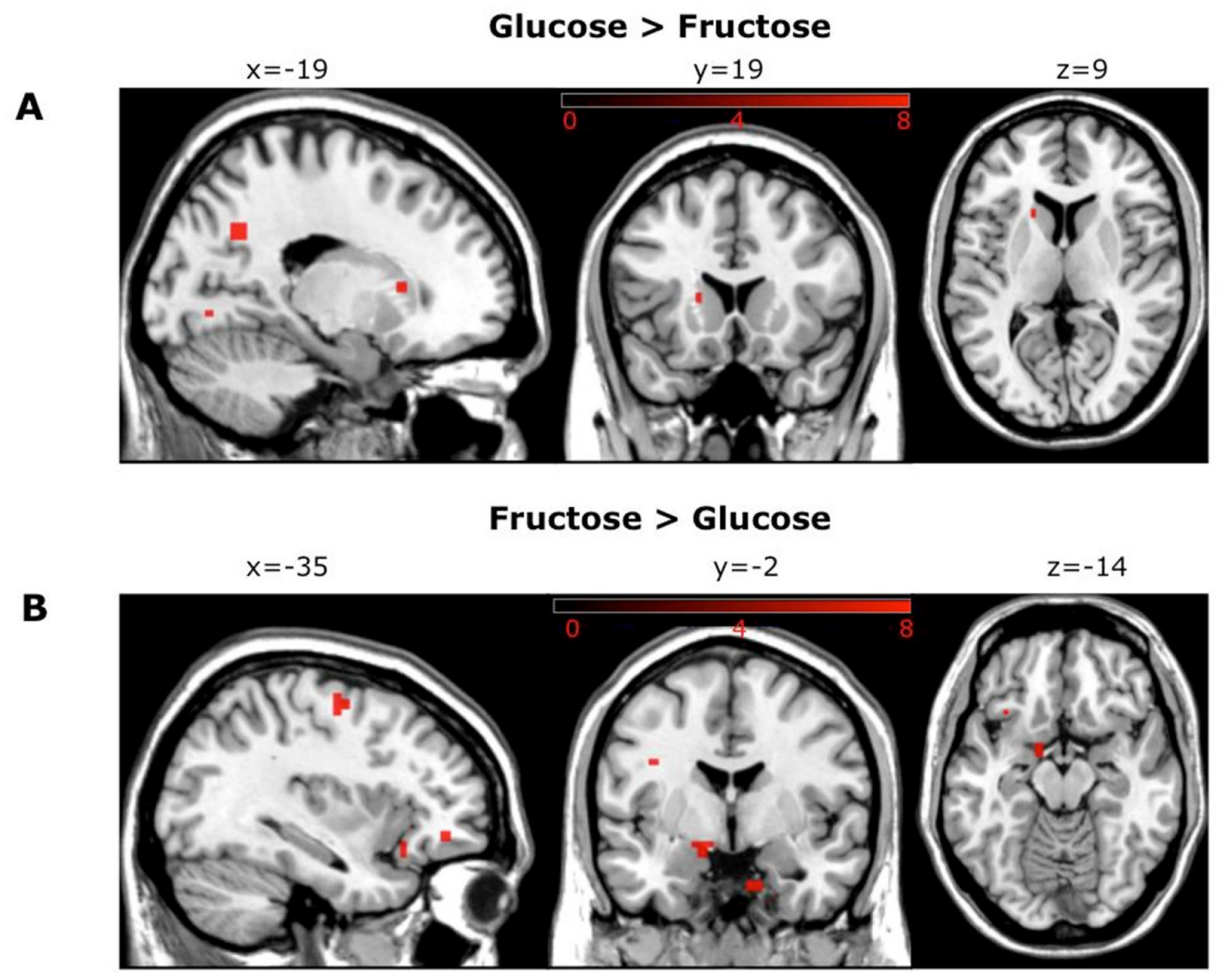
Differences in functional resting state connectivity to the basal ganglia/limbic network between glucose and fructose administration. (A) Dual regression to the basal ganglia/limbic network demonstrates a glucose-induced increase in rsFC of the left caudatus (x = -17, y = 18, z = 8), left putamen (x = -34, y = -18, z = -8), precuneus (x = -18, y = -60, z = 32) and lingual gyrus (x = -18, y = -73, z = -3) relative to fructose (p = 0.02 uncorrected) (B) Fructose increased rsFC of left amygdala (x = -14, y = -3, z = -14), left hippocampus (x = -18, y = -4, z = -24), right (para)-hippocampus (x = 11, y = 0, z = -32), OFC (x = -33, y = 23, z = -16) and precentral gyrus (x = -34, y = -8, z = 57) compared with glucose (p = 0.02 uncorrected).

After glucose, increased rsFC of the left caudatus and putamen, precuneus and lingual gyrus was found, relative to fructose. After fructose, increased rsFC of the left amygdala, left hippocampus, right parahippocampus, OFC and precentral gyrus to the basal ganglia/limbic network was found relative to glucose (Fig 5).

### Correlations between behavioral, physiological and neural effects

**Fig 6:**
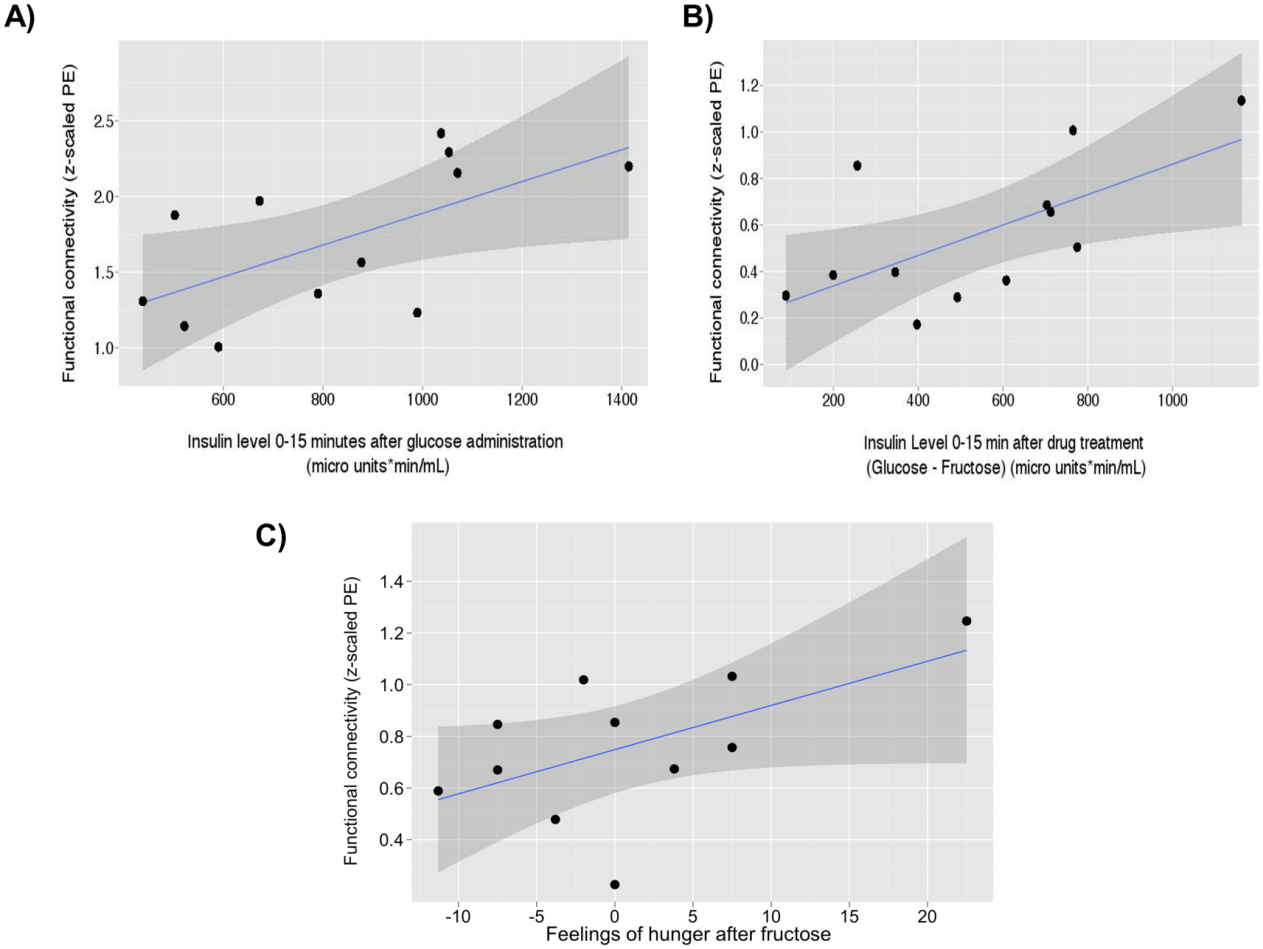
(A) Correlations between resting state functional connectivity, insulin release and subjective appetite perception. In relation to placebo, the glucose-induced increase in functional connectivity strength (parameter estimates; PE) within the basal ganglia/limbic network correlated positively with the glucose-induced insulin release (μU/mL) (r = 0.62, p = 0.03). (B) Relative to fructose, the glucose-induced increase in functional connectivity strength within basal ganglia/limbic network correlated positively with and the glucose-induced insulin release (r = 0.65, p = 0.02). (C) In relation to placebo, the fructose-induced increase in functional connectivity strength within the basal ganglia/limbic network correlated positively at trend level with the fructose-induced feeling of hunger (r = 0.57, p = 0.069).

Exploratory correlation analysis showed that the increased rsFC within the basal ganglia/limbic network induced by glucose relative to placebo correlated positively with the insulin level after glucose ingestion (r = 0.62, p = 0.03), as the glucose-induced effect on rsFC relative to fructose (r = 0.65, p = 0.02; Fig 6A and 6B, uncorrected for multiple testing). Moreover, there was a trend between the fructose-induced effect within the basal ganglia network relative to placebo and the fructose-induced feelings of hunger (r = 0.57, p = 0.069; Fig 6C, uncorrected for multiple testing). No other significant correlations between resting state data, behavioral and physiological parameters were found.

## Discussion

The present study explored whether the different effects of acute glucose and fructose ingestion could also be observed in the neural system by using resting state functional MRI. We first showed that glucose increased feelings of fullness and decreased prospective food consumption compared with fructose. Secondly, glucose ingestion induced significantly greater elevations in plasma glucose, insulin, GLP-1 and GIP concentrations compared to fructose. Thirdly, glucose relative to fructose ingestion increased rsFC of the left caudatus and putamen, precuneus and lingual gyrus, whereas fructose relative to glucose increased rsFC of the left amygdala, left hippocampus, right parahippocampus, orbitofrontal cortex and precentral gyrus within the basal ganglia/limbic network. Finally and perhaps most interestingly, we found that the increased rsFC after glucose correlated positively with the glucose-induced increase in insulin relative to fructose, which suggests a relationship between physiological and neural effects.

Fructose consumption from industrially produced foods is increasing worldwide, and this may be accompanied by adverse metabolic consequences [35,36]. In our study, there were only minimal changes in satiation hormones after fructose ingestion, which is in line with previous studies [18,19]. Various trials have been carried out to answer the question of whether fructose consumption has the same or even stronger satiating effects than glucose or on the contrary results in increased food intake [37,38]. In animal studies fructose increased subsequent food intake—an orexigenic effect—while glucose led to satiation [39]. In humans, fructose triggers minimal release of satiation hormones and adiposity signals combined with attenuated postprandial suppression of ghrelin; this suggests that food intake may be stimulated by fructose intake as well [37]. In addition, gastric emptying—another important satiety signal—is decelerated by glucose ingestion, but apparently not by fructose ingestion [40]. The failure of fructose to influence appetite perception is consistent with this. Of note, placebo values lie in between fructose and glucose (Fig 2) as if fructose led to less satiation than placebo or to put it the other way round: as if fructose might even promote hunger. However, no statistical significance could be reached between the respective carbohydrate and placebo and additional studies with larger sample size are needed to confirm this finding. Previous fMRI studies investigating hypothalamic reactions have compared intravenous with oral glucose administration and showed that the effect is much stronger after oral ingestion, which emphasizes the importance of gut-signalling [41]. A recent resting state functional MRI investigation found that glucose ingestion increased FC between the hypothalamus, thalamus and striatum, whereas fructose increased FC between the hypothalamus and thalamus but not the striatum [42]. Although we used a different methodological approach—with a focus on the basal ganglia/limbic network rather than on the hypothalamus—our results are in line with these findings: glucose but not fructose increased FC of the caudatus and putamen to the basal ganglia/limbic network. Interestingly, Page et al. found a negative correlation between striatal blood flow and the glucose-induced change in insulin levels, which suggests that anorexigenic peptides such as insulin decrease the sensitivity of the brain reward system to food reward [5]. In our study, we found that the increased striatal connectivity to the basal ganglia network after glucose relative to either fructose or water administration correlated positively with the glucose-induced insulin level. This seems to be at odds with Page et al.’s finding of an inverse relationship between insulin release and striatal activity [13]. However, besides the fact that they used equicaloric doses of fructose and glucose (75g vs. 75g), there is an important temporal discrepancy in the methodical design of the studies: While Page et al. employed a 60-minute post-ingestion acquisition period of pulsed arterial spin labeling and functional MRI sequences, we examined our subjects directly after glucose and fructose ingestion with one resting state sequence. Our rationale for this time frame is that the peak of hormone release is found at about 10–15 minutes post ingestion and fructose is rapidly converted to glucose in the liver so that the time window for distinguishing fructose from glucose effects in the brain is very short [39]. More importantly, 75g of fructose is a very high load and can cause adverse events even in healthy subjects: 25g is the highest load of fructose absorbed by healthy volunteers and a fructose load of 50g induces unspecific results in 40–60% of individuals [43,44]. Therefore, we used 75g of glucose (as in a standard glucose tolerance test) and 25g of fructose (the highest absorbable load).

The acute rewarding effect of food intake is probably mediated by dopamine [5]. Indeed, in humans, ingestion of palatable food has been shown to release dopamine in the dorsal striatum in proportion to the self-reported level of pleasure [45]. This striatal rewarding effect corresponds to the subjective effects of acute drug intake that are mediated by reinforcement signals in the striatum, and may imply that there are overlaps in the brain circuits underlying food intake and drug-intake [46]. We propose that the acute increase in striatal connectivity to the basal ganglia network after glucose ingestion may mediate a state of satiation, which was modulated by insulin release. This interpretation is consistent with a recent study that showed that glucose ingestion in insulin-sensitive subjects leads to significantly greater dopamine release in the brain reward regions than in insulin-resistant subjects [47].

In contrast, fructose did not increase striatal connectivity to the basal ganglia network as seen after glucose in this study, but increased FC of the amygdala, hippocampus and OFC (orbito-frontal cortex) compared with glucose. One may ask whether this neural pattern corresponds to the fact that food intake in humans might even be stimulated rather than blocked by fructose intake [37]. The motivation for a continued food intake after chronic fructose exposure may be driven by down regulation of dopaminergic D2 receptors in reward-sensitive pathways [16,17]. The decreased activation of dopaminergic targets by the current food consumption might lead to overconsumption to compensate for the weak dopamine signals [48]. Indeed, OFC activity has been positively associated with food craving in lean subjects [49]. Furthermore, it has been shown that when lean individuals were asked to inhibit their food craving when exposed to cues, their activity in the amygdala, hippocampus and OFC decreased [50]. Considering the relevance of these regions in drug addiction, evidence has consistently demonstrated FC between the OFC and amygdala during withdrawal-induced craving [51]. The OFC appears to be important for stress-induced reinstatement of drug seeking, i.e., it drives the motivation for new drug intake, during which it is reciprocally connected with the amygdala [52,53]. Furthermore, OFC activation is correlated with hunger ratings during presentation of food items [8]. An infusion of ghrelin, which heightens appetite, increases the amygdala response to food images [9]. Both fasting and ghrelin administration also increased hippocampus activation to food pictures [54], whereas insulin decreases OFC activity [12]. In this case, fructose did not stimulate insulin release and did not suppress ghrelin release as shown in previous studies by our group [18] and failed to influence appetite perception; we therefore suggest that the fructose-induced increase in OFC, amygdala and hippocampus FC to the basal ganglia network indicates a drive for renewed food intake. This interpretation is further supported by the positive correlation between the effect of fructose on OFC connectivity and subjective feelings of hunger. We propose that fructose stimulates dopaminergic regions that mediate the reward and reinforcement effect of food intake insufficiently. However, this is clearly speculative at the present time and many more studies are needed to support such an interpretation.

One limitation of this study is the explicit focus on the basal ganglia/limbic network without considering its interaction with the homeostatic system after glucose and fructose administration; this point should be addressed in future studies. However, we explicitly focused on this network as it includes the key region for reward, i.e. the striatum. Another point of contention may be that our analysis only revealed uncorrected results in terms of appetite perception and resting state functional connectivity. The sample size in this pilot study was modest as these controlled repeated measures within-subject design studies are logistically demanding. Nevertheless, larger sample sizes are required to draw more statistically valid inferences. Furthermore, both sexes should be included and effects of low, equicaloric doses of fructose and glucose should be examined as well (e.g. 25g glucose and 25g fructose).

In conclusion, to the best of our knowledge, this is the first resting state functional MRI study addressing the effect of acute glucose and fructose ingestion. Our findings provide preliminary evidence that fructose modulates FC to the basal ganglia network in a different manner to glucose. These distinct effects may help explain why and how glucose and fructose have different behavioral and physiological effects.

More imaging research with larger sample sizes is needed to understand how glucose and fructose modulate the neural system.

## Supporting Information

S1 Protocol Trial study Protocol. (PDF)

S1 Table Treatment differences of resting state functional connectivity to the basal ganglia network relative to placebo. (PDF)

S1 Text TREND checklist. (TIFF)

S2 Text TREND checklist. (TIFF)

S3 Text TREND checklist. (TIFF)

## References

[1] Aziz Q (2012) Brain-gut interactions in the regulation of satiety: new insights from functional brain imaging. Gut 61: 1521–1522. 22661493 10.1136/gutjnl-2012-302368

[2] Schmidt A, Hammann F, Wölnerhanssen B, Meyer-Gerspach AC, Drewe J, Beglinger C, et al (2014) Green tea extract enhances parieto-frontal connectivity during working memory processing. Psychopharmacology (Berl) 231: 3879–3888. 10.1007/s00213-014-3526-1 24643507 PMC4159594

[3] Berthoud HR (2004) Neural control of appetite: cross-talk between homeostatic and non-homeostatic systems. Appetite 43: 315–317. 15527935 10.1016/j.appet.2004.04.009

[4] Morton GJ, Cummings DE, Baskin DG, Barsh GS, Schwartz MW (2006) Central nervous system control of food intake and body weight. Nature 443: 289–295. 16988703 10.1038/nature05026

[5] Volkow ND, Wang GJ, Baler RD (2011) Reward, dopamine and the control of food intake: implications for obesity. Trends Cogn Sci 15: 37–46. 10.1016/j.tics.2010.11.001 21109477 PMC3124340

[6] Volkow ND, Wang GJ, Tomasi D, Baler RD (2013) The addictive dimensionality of obesity. Biol Psychiatry 73: 811–818. 10.1016/j.biopsych.2012.12.020 23374642 PMC4827347

[7] Kenny PJ (2011) Reward mechanisms in obesity: new insights and future directions. Neuron 69: 664–679. 10.1016/j.neuron.2011.02.016 21338878 PMC3057652

[8] Morris JS, Dolan RJ (2001) Involvement of human amygdala and orbitofrontal cortex in hunger-enhanced memory for food stimuli. J Neurosci 21: 5304–5310. 11438606 10.1523/JNEUROSCI.21-14-05304.2001 PMC6762827

[9] Malik S, McGlone F, Bedrossian D, Dagher A (2008) Ghrelin modulates brain activity in areas that control appetitive behavior. Cell Metab 7: 400–409. 10.1016/j.cmet.2008.03.007 18460331

[10] Farooqi IS, Bullmore E, Keogh J, Gillard J, O'Rahilly S, Fletcher PC (2007) Leptin regulates striatal regions and human eating behavior. Science 317: 1355 17690262 10.1126/science.1144599 PMC3838941

[11] Coll AP, Farooqi IS, O'Rahilly S (2007) The hormonal control of food intake. Cell 129: 251–262. 17448988 10.1016/j.cell.2007.04.001 PMC2202913

[12] Kullmann S, Frank S, Heni M, Ketterer C, Veit R, Haring HU, et al (2013) Intranasal insulin modulates intrinsic reward and prefrontal circuitry of the human brain in lean women. Neuroendocrinology 97: 176–182. 10.1159/000341406 22922661

[13] Figlewicz DP, Sipols AJ (2010) Energy regulatory signals and food reward. Pharmacol Biochem Behav 97: 15–24. 10.1016/j.pbb.2010.03.002 20230849 PMC2897918

[14] Havel PJ (2005) Dietary fructose: implications for dysregulation of energy homeostasis and lipid/carbohydrate metabolism. Nutr Rev 63: 133–157. 15971409 10.1301/nr.2005.may.133-157

[15] Malik VS, Popkin BM, Bray GA, Despres JP, Willett WC, Hu FB(2010) Sugar-sweetened beverages and risk of metabolic syndrome and type 2 diabetes: a meta-analysis. Diabetes Care 33: 2477–2483. 10.2337/dc10-1079 20693348 PMC2963518

[16] Lustig RH (2013) Fructose: it's "alcohol without the buzz". Adv Nutr 4: 226–235. 10.3945/an.112.002998 23493539 PMC3649103

[17] Spangler R, Wittkowski KM, Goddard NL, Avena NM, Hoebel BG, Leibowitz SF (2004) Opiate-like effects of sugar on gene expression in reward areas of the rat brain. Brain Res Mol Brain Res 124: 134–142. 15135221 10.1016/j.molbrainres.2004.02.013

[18] Steinert RE, Frey F, Topfer A, Drewe J, Beglinger C (2011) Effects of carbohydrate sugars and artificial sweeteners on appetite and the secretion of gastrointestinal satiety peptides. Br J Nutr 105: 1320–1328. 10.1017/S000711451000512X 21255472

[19] Teff KL, Elliott SS, Tschop M, Kieffer TJ, Rader D, Heiman M, et al (2004) Dietary fructose reduces circulating insulin and leptin, attenuates postprandial suppression of ghrelin, and increases triglycerides in women. J Clin Endocrinol Metab 89: 2963–2972. 15181085 10.1210/jc.2003-031855

[20] Rolls BJ, Kim S, Fedoroff IC (1990) Effects of drinks sweetened with sucrose or aspartame on hunger, thirst and food intake in men. Physiol Behav 48: 19–26. 2236270 10.1016/0031-9384(90)90254-2

[21] Biswal BB, Mennes M, Zuo XN, Gohel S, Kelly C, Smith SM, et al (2010) Toward discovery science of human brain function. Proc Natl Acad Sci U S A 107: 4734–4739. 10.1073/pnas.0911855107 20176931 PMC2842060

[22] Fox MD, Snyder AZ, Vincent JL, Corbetta M, Van Essen DC, Raichle ME (2005) The human brain is intrinsically organized into dynamic, anticorrelated functional networks. Proc Natl Acad Sci U S A 102: 9673–9678. 15976020 10.1073/pnas.0504136102 PMC1157105

[23] Beckmann CF, DeLuca M, Devlin JT, Smith SM (2005) Investigations into resting-state connectivity using independent component analysis. Philos Trans R Soc Lond B Biol Sci 360: 1001–1013. 16087444 10.1098/rstb.2005.1634 PMC1854918

[24] Smith SM, Fox PT, Miller KL, Glahn DC, Fox PM, Mackay CE, et al (2009) Correspondence of the brain's functional architecture during activation and rest. Proc Natl Acad Sci U S A 106: 13040–13045. 10.1073/pnas.0905267106 19620724 PMC2722273

[25] Cole DM, Beckmann CF, Searle GE, Plisson C, Tziortzi AC, Nichols TE, et al (2012) Orbitofrontal connectivity with resting-state networks is associated with midbrain dopamine D3 receptor availability. Cereb Cortex 22: 2784–2793. 10.1093/cercor/bhr354 22186675

[26] Moussa MN, Steen MR, Laurienti PJ, Hayasaka S (2012) Consistency of network modules in resting-state FMRI connectome data. PLoS One 7: e44428 10.1371/journal.pone.0044428 22952978 PMC3432126

[27] Sesack SR, Grace AA (2010) Cortico-Basal Ganglia reward network: microcircuitry. Neuropsychopharmacology 35: 27–47. 10.1038/npp.2009.93 19675534 PMC2879005

[28] Flint A, Raben A, Blundell JE, Astrup A (2000) Reproducibility, power and validity of visual analogue scales in assessment of appetite sensations in single test meal studies. Int J Obes Relat Metab Disord 24: 38–48. 10702749 10.1038/sj.ijo.0801083

[29] Jenkinson M, Bannister P, Brady M, Smith S (2002) Improved optimization for the robust and accurate linear registration and motion correction of brain images. Neuroimage 17: 825–841. 12377157 10.1016/s1053-8119(02)91132-8

[30] Smith SM (2002) Fast robust automated brain extraction. Hum Brain Mapp 17: 143–155. 12391568 10.1002/hbm.10062 PMC6871816

[31] Filippini N, MacIntosh BJ, Hough MG, Goodwin GM, Frisoni GB, Smith SM, et al (2009) Distinct patterns of brain activity in young carriers of the APOE-epsilon4 allele. Proc Natl Acad Sci U S A 106: 7209–7214. 10.1073/pnas.0811879106 19357304 PMC2678478

[32] Nichols TE, Holmes AP (2002) Nonparametric permutation tests for functional neuroimaging: a primer with examples. Hum Brain Mapp 15: 1–25. 11747097 10.1002/hbm.1058 PMC6871862

[33] Smith SM, Nichols TE (2009) Threshold-free cluster enhancement: addressing problems of smoothing, threshold dependence and localisation in cluster inference. Neuroimage 44: 83–98. 10.1016/j.neuroimage.2008.03.061 18501637

[34] Rowland M, Tozer T.N. (1989) Clinical Pharmacokinetics—Concepts and Applications. Published by Lea & Febiger (Malvern, PA) Second edition.

[35] Malik VS, Hu FB (2012) Sweeteners and Risk of Obesity and Type 2 Diabetes: The Role of Sugar-Sweetened Beverages. Curr Diab Rep. 10.1007/s11892-012-0259-6 22289979

[36] Le MT, Frye RF, Rivard CJ, Cheng J, McFann KK, Segal MS, et al (2012) Effects of high-fructose corn syrup and sucrose on the pharmacokinetics of fructose and acute metabolic and hemodynamic responses in healthy subjects. Metabolism 61: 641–651. 10.1016/j.metabol.2011.09.013 22152650 PMC3306467

[37] Moran TH (2009) Fructose and satiety. J Nutr 139: 1253S–1256S. 10.3945/jn.108.097956 19403706 PMC6459054

[38] Moyer AE, Rodin J (1993) Fructose and behavior: does fructose influence food intake and macronutrient selection? Am J Clin Nutr 58: 810S–814S. 8213614 10.1093/ajcn/58.5.810S

[39] Cha SH, Wolfgang M, Tokutake Y, Chohnan S, Lane MD (2008) Differential effects of central fructose and glucose on hypothalamic malonyl-CoA and food intake. Proc Natl Acad Sci U S A 105: 16871–16875. 10.1073/pnas.0809255105 18971329 PMC2579345

[40] Guss JL, Kissileff HR, Pi-Sunyer FX (1994) Effects of glucose and fructose solutions on food intake and gastric emptying in nonobese women. Am J Physiol 267: R1537–1544. 7810763 10.1152/ajpregu.1994.267.6.R1537

[41] Smeets PA, Vidarsdottir S, de Graaf C, Stafleu A, van Osch MJ, Viergever MA, et al (2007) Oral glucose intake inhibits hypothalamic neuronal activity more effectively than glucose infusion. Am J Physiol Endocrinol Metab 293: E754–758. 17566114 10.1152/ajpendo.00231.2007

[42] Page KA, Chan O, Arora J, Belfort-Deaguiar R, Dzuira J, Roehmholdt B, et al (2013) Effects of fructose vs glucose on regional cerebral blood flow in brain regions involved with appetite and reward pathways. JAMA 309: 63–70. 10.1001/jama.2012.116975 23280226 PMC4076145

[43] Rao SS, Attaluri A, Anderson L, Stumbo P (2007) Ability of the normal human small intestine to absorb fructose: evaluation by breath testing. Clin Gastroenterol Hepatol 5: 959–963. 17625977 10.1016/j.cgh.2007.04.008 PMC1994910

[44] Frieling T, Kuhlbusch-Zicklam R, Kalde S, Heise J, Hulsdonk A, Kreysel C (2011) Fructose malabsorption: how much fructose can a healthy subject tolerate? Digestion 84: 269–272. 10.1159/000329570 21952629

[45] Small DM, Jones-Gotman M, Dagher A (2003) Feeding-induced dopamine release in dorsal striatum correlates with meal pleasantness ratings in healthy human volunteers. Neuroimage 19: 1709–1715. 12948725 10.1016/s1053-8119(03)00253-2

[46] Volkow ND, Wang GJ, Tomasi D, Baler RD (2013) Obesity and addiction: neurobiological overlaps. Obes Rev 14: 2–18. 10.1111/j.1467-789X.2012.01031.x 23016694 PMC4827343

[47] Wang GJ (2013) Peripheral insulin resistance affects brain dopaminergic signaling after glucose ingestion SNMMI's 60th Annual Meeting. Vancouver, British Columbia. pp. Available: http://interactive.snm.org/index.cfm?PageID=12685

[48] Stice E, Spoor S, Bohon C, Small DM (2008) Relation between obesity and blunted striatal response to food is moderated by TaqIA A1 allele. Science 322: 449–452. 10.1126/science.1161550 18927395 PMC2681095

[49] Wang GJ, Volkow ND, Telang F, Jayne M, Ma J, Rao M, et al (2004) Exposure to appetitive food stimuli markedly activates the human brain. Neuroimage 21: 1790–1797. 15050599 10.1016/j.neuroimage.2003.11.026

[50] Wang GJ, Volkow ND, Telang F, Jayne M, Ma Y, Pradhan K, et al (2009) Evidence of gender differences in the ability to inhibit brain activation elicited by food stimulation. Proc Natl Acad Sci U S A 106: 1249–1254. 10.1073/pnas.0807423106 19164587 PMC2633545

[51] Koob GF, Volkow ND (2010) Neurocircuitry of addiction. Neuropsychopharmacology 35: 217–238. 10.1038/npp.2009.110 19710631 PMC2805560

[52] Lasseter HC, Ramirez DR, Xie X, Fuchs RA (2009) Involvement of the lateral orbitofrontal cortex in drug context-induced reinstatement of cocaine-seeking behavior in rats. Eur J Neurosci 30: 1370–1381. 10.1111/j.1460-9568.2009.06906.x 19769591 PMC2758926

[53] Schoenbaum G, Shaham Y (2008) The role of orbitofrontal cortex in drug addiction: a review of preclinical studies. Biol Psychiatry 63: 256–262. 17719014 10.1016/j.biopsych.2007.06.003 PMC2246020

[54] Goldstone AP, Prechtl CG, Scholtz S, Miras AD, Chhina N, Durighel G, et al (2014) Ghrelin mimics fasting to enhance human hedonic, orbitofrontal cortex, and hippocampal responses to food. Am J Clin Nutr 99: 1319–1330. 10.3945/ajcn.113.075291 24760977 PMC6410902

