# Supplementary material for: Dissociable Behavioral, Physiological and Neural Effects of Acute Glucose and Fructose Ingestion: A Pilot Study
Source: PLoS One. 2015 Jun 24;10(6):e0130280. doi: 10.1371/journal.pone.0130280 (PMC4481317; doi:10.1371/journal.pone.0130280)
Supplement: S1 Protocol — (PDF) [file pone.0130280.s001.pdf]

Effects of Metabolic Surgery On the Central Satiety System, Gastrointestinal Motility and Taste Receptors:

Project 1: Functional MRI Glucose/Fructose treatment in morbidly obese patients

Prospective Cohort Study of Morbidly Obese patients
Before and after Bariatric Surgery

Universitätsspital Basel

Gastroenterologie und Hepatologie

Bettina Wölnerhanssen, MD
University Hospital Basel

Date 1. Version: 11.10.2012

Signature: ________________________
(Dr. B. Wölnerhanssen)

Date 2. Version: 29.11.2012

Date 3 Version: 15.8.2013

CONTENTS

INTRODUCTION **4**
Morbid Obesity and Diabetes 4
Satiety System: Gut Hormones 4
Taste Receptors 5

HYPOTHESES **6**

AIMS **6**

GENERAL CONSIDERATIONS **7**
Physical health 7
Informed consent 7
Ethical standards 7
Data protection
Insurance 8

PROJECTS
Project 1: Functional MRI Glucose/Fructose treatment in morbidly obese patients **8**
Background 8
Hypotheses 9
Methods 9
Study design 9
Study duration and study site 9
Recruitment and screening 10
Subjects 10
Study protocol 11
Overall design 11
Experimental procedure 11
Data analysis 12
Risks and benefits 12
Compensation 12
Experimental design 13

REFERENCES **25**

INTRODUCTION

Morbid obesity and Diabetes

Obesity has become one of the greatest public health challenges in the 21st century, with an epidemic prospect that > 50% of the world's adult population will be overweight and obese by 2030[1]. Of special interest in this context is diabetes – the most expensive co-morbidity; a disease which in 80% is associated with overweight[2]. Bariatric surgery is currently the only effective treatment for morbid obesity and its co-morbidities, especially diabetes[3-5]. Improvement of glucose metabolism after bariatric surgery is impressive, as we could show in our own prospective trials[6]. Though there are some hypotheses how glucose metabolism ameliorates early after surgery (before weight-loss occurs), the exact mechanisms are not known. One of our on-going studies comparing morbidly obese non-diabetic to diabetic patients before and after surgery suggest that the mechanism underlying glucose intolerance in these two groups is not the same (*Wölnerhanssen et al,* unpublished data). Studies in this field can help to better understand the mechanisms of diabetes in general and in the morbidly obese in particular.

The pathophysiological background of morbid obesity is not yet clear, nor is the physiology behind appetite, satiety regulation and weight-maintenance fully understood. Surgery has been proven to be effective in terms of weight-loss and reduction in co-morbidities. However, the exact mechanisms underlying different bariatric procedures are yet unknown. Progress in treatment other than surgery critically depends on high-quality clinical research in this field. At least theoretically, knowing more about morbid obesity and mechanisms of bariatric surgery could lead to interesting targets for treatment regimens in the future. Morbidly obese patients treated by different bariatric procedures with different anatomical changes of the GI-tract offer a great opportunity to study the pathophysiology behind morbid obesity.

Satiety system: gut hormones

Various gut hormones regulate food intake and some even show direct effects on glucose metabolism. One promising approach is the development of drugs based on gastrointestinal (GI) satiety peptides such as glucagon-like peptide-1 (GLP-1). Its anorectic action is of great clinical interest as the eating-inhibitory effect is not only seen in healthy subjects, but is preserved in obese and/or diabetic persons[7-9]. Understanding the exact mechanisms by which GLP-1 inhibits eating is crucial in order to convert its anorectic action into useful, safe and effective drugs. After eating, the brain senses a biochemical change and then signals satiation, but the precise sequence of events has not yet been determined. Even for established physiological systems like glucose-insulin regulation, the timing of interaction between hormonal processes and neural events is inferred mostly from blood sampling studies. Recently, neuro-imaging studies have provided in vivo information about the neuro-anatomical correlates of the regulation of energy intake. Temporal orchestration of such systems is, however, crucial to the integration of neural and hormonal signals that control eating behavior. In a landmark paper demonstrating eating-related neural activity in the brain, the response was interacting with an internal signal, the plasma insulin. Oral glucose is a potent stimulant for insulin release modified by the incretin hormone GLP-1. Furthermore exogenous administration of GLP-1 to healthy humans or to diabetic patients dose-dependently reduces food intake and induces satiety suggesting that the peptide could be one of the gut players interacting with the brain[10, 11].

Healthy, normal-weight people show a postprandial peak of GLP-1 secretion after 15-30 min. In contrast in morbidly obese patients, postprandial GLP-1 secretion is blunted. After bariatric surgery, dynamics of GLP-1 secretion are restored[12].

In mice, functional MRI after GLP-1 administration showed reduced signal intensity in the targets PVN (paraventricular nucleus) and VMH (ventromedial hypothalamus)[13, 14] In humans, functional MRI in correlation with GLP-1 levels has recently been carried out on healthy volunteers at the Imperial College of London: Infusion of GLP-1 led to reduction of brain activity across all selected ROI's (regions of interest), the greatest change seen in the right insula[15]. Thus, the GLP-1 targets in the human brain are known. However, so far studies on morbidly obese before and after surgery have not yet been carried out. This offers an excellent possibility to investigate neuro-anatomical correlates of the regulation of food intake and examine gut-brain signaling, with special focus on the obese.
The results of our recent studies document that glucose as well as fatty acids are both potent secretagogues for GLP-1 secretion, whereas artificial sweeteners do not induce a GLP-1 response. The effect is most likely mediated in large part by a heterodimeric sweet taste receptor, T1R2/T1R3. Lactisole©, a broad-acting sweet taste receptor antagonist, suppresses the sweet taste of sugars, protein sweeteners, and artificial sweeteners in vitro[16] and has been approved for use in food as flavoring agent. Due to its inhibitory effects on the sweet receptor, lactisole is an excellent tool to better understand the regulatory role of this receptor in the control of glucose metabolism.

Taste receptors

Enteroendocrine cells (EECs) in gut villi are highly specific cells, which in total, constitute the largest endocrine organ. They release a series of hormones including cholecystokinin (I-cells), ghrelin (X-cells), gastrin (G-cells), glucose-dependent-insulinotropic peptide (GIP) (K-cells), glucagon-like peptides (GLPs) and peptide YY (PYY) (L-cells) [17, 18]. The products are released from secretory granules into the extracellular fluids to enter the circulation to act on distant targets or to interact locally with intrinsic and extrinsic (predominantly vagal) afferent neurons[19, 20]. In recent years, it has been speculated that taste receptors like those known from the tongue might be involved in the receptive function within the intestine[21, 22]. In fact, in 1996, taste receptor-like cells were first identified in the rat gut by the expression of α-gustducin [23]. Subsequent studies demonstrated the presence of several nutrient responsive G-protein-coupled receptors (GPCRs) for bitter taste (T2Rs) and amino acid, umami and sweet taste (T1Rs) both in human and rodent gut, including distribution and co-localization with L-cells expressing GLP-1 or PYY [24-27]. In addition, studies in mice using knockout models for α-gustducin (α -gust-/-) or T1R3 (T1R3-/-) (part of the sweet-responsive heterodimer T1R2/T1R3) revealed deficiencies in glucose-stimulated secretion of GLP-1, and the regulation of plasma insulin and glucose[28]. Moreover, both carbohydrate sugars and artificial sweeteners were capable of stimulating the secretion of GLP-1 from EEC lines, and specific sweet taste receptor antagonists (lactisole or gurmarin) blocked the secretion of artificial sweetener-stimulated gut peptide secretion[28, 29]. Taken together, these data provide extensive evidence for the involvement of α-gustducin-coupled taste receptors in nutrient-stimulated peptide secretion. Although these basic chemosensory mechanisms have been demonstrated in several in vitro and animal studies, human data are still sparse. Recently, we were able to show co-localization of T1R3 and α-gustducin with GLP-1 in human duodenal and colonic biopsy specimens using immunohistochemistry[30]. By use

of quantitative real time RT-PCR (qPCR) we could show, that the level of mRNA-expression was equally distributed in all gut segments for T1R3; in contrast α-gustducin was found to be expressed predominately in the duodenum[30]. However, these data were obtained from healthy, normal-weight patients and unfortunately stomach tissue was not involved.

HYPOTHESES

- Obesity is associated with changes in taste reception, especially desensitization of sweet reception and probably altered perception of umami. We expect to see these differences:

  a) on the level of the *central nervous system* by means of fMRI (decreased brain activation after glucose administration in the obese), b) on the level of *taste receptors* in gastric and intestinal tissue (e.g. decreased number of T1R3) as well as c) on the level of *hormonal release* (e.g. decreased GLP-1 release after glucose stimulation).

- Bariatric surgery leads to changes in gastrointestinal motility, depending on the chosen type of procedure (bypass vs. sleeve gastrectomy).

AIMS

The molecular and cellular mechanism(s) by which the biochemical composition of ingested nutrients are sensed within the intestinal lumen by enteroendocrine cells, the signaling pathways to the CNS, and the factors that modulate food intake, are poorly defined. The key ideas of this proposal are based upon world leading current neuro-imaging work in Basel that has started to map out the matrix of activation of the central nervous system upon activation of these neuro-endocrine pathways. The aim of this project is to learn more about the physiology of appetite and satiety regulation in humans by examining healthy volunteers and to correlate these findings with data from morbidly patients in order to better understand the pathophysiology behind morbid obesity. Pre- and postoperative studies will help to better understand mechanism(s) underlying bariatric procedures.

Key questions are:

1) Enlighten the gut brain axis

2) Describe interactions of different substrates on brain activity and gut hormones

3) Clarify effects of bariatric surgery on hepatic and pancreatic fat distribution

GENERAL CONSIDERATIONS

Physical health

All subjects will be examined by a research physician. Basic health will be ensured by general medical examination including medical history, physical examination and blood chemistry and hematology. Anthropometric measurements including weight, height, body mass index (BMI), as well as heart rate and blood pressure will be recorded for all participants.

Informed consent

All subjects will be informed about the study they participate in, both verbally and by the approved written consent form regarding study procedures. The investigator and the subject will both personally sign and date the consent form as confirmation of consent. All studies will be approved by the Ethics Committee of the University of Basel Participation is voluntary. Participants do not profit directly from the studies.

Ethical Standards

All studies will be performed in accordance with the Declaration of Helsinki, International Conference on Harmonization, Guidelines for Good Clinical Practice (GCP) and standard operating procedures of the CRC/CTU of the University Hospital of Basel. Trained study personnel will place tubes and venous accesses, administer the perfusions and collect blood samples. Subjects will be supervised by trained study personnel during the entire test sessions, backed-up by study physicians. All studies will be done in the Phase 1 Research Unit of the University Hospital. To make sure that quality requirements are fulfilled, the Ethical Committee is empowered to audit our institution.

Data protection

All information will be treated in confidence. At any time, participants have the right to gain access to their results. All data is anonymized. For control purposes the Ethical Committee and the authorities can request to undertake inspections and gain access to original data. Biopsies will only be used for the described study and after that any tissue remnants will be destroyed (no biobank will be established).

Insurance

Subjects will be insured for this trial by Gerlach Insurance group via the University Hospital. The principle investigator will be responsible for the initiation of all necessary action in the case of harm to a subject. Studies carried out at St. Claraspital will be insured via their insurance partner.

Functional MRI after fructose and glucose-stimulation in morbidly obese patients

Background

Fructose is a monosaccharide naturally found in honey, flowers and fruits. High-fructose corn syrup (HFCS) is a mixture of glucose and fructose and is found in many soft drinks. Fructose is believed to have adversary effects on diabetes and weight gain[31]. A fructose-enriched diet might lead to insulin resistance and obesity and therefore increase in fructose consumption is seen as a risk factor for metabolic syndrome[32]. Glucose is the only potent secretagogue of the release of satiety peptides such as GLP-1, PYY and inhibitor of ghrelin. In contrast, fructose infusion in healthy, normal-weight subjects doesn't affect plasma concentrations of the above mentioned peptides to the same extent as equisweet loads of glucose[16]. Nevertheless, fructose leads to prolonged satiety[16]. The pathway leading to satiety must be different from the known pathway of glucose. In rat experiments, obese rats were more interested in fructose than lean controls (preference for higher concentration) and after gastric bypass increased lick responses were alleviated[33]. There are no studies describing fructose effects on gut hormones, satiety and central effects before and after bariatric surgery in humans.
In previous studies, 50-75g of glucose showed reliable GLP-1 release and brain activation. For fructose, no such data exists.

Ongoing study: From March 2012 till November 2012 we are examining 12 healthy, normal-weight volunteers by functional MRI and intragastric administration of a glucose solution with or without the sweet taste receptor blocker lactisole© , 25g fructose and 75g glucose with the GLP-1 antagonist exendin 9-39 in collaboration with Prof. S. Borgwardt. This offers an excellent possibility to investigate the neuro-anatomical correlates of the regulation of food intake and examine gut-brain signaling in healthy subjects. We believe that glucose in the gut affects the brain by means of a cascade, which is triggered by sweet taste receptor activation and mediated by the vagus nerve. The sweet taste receptor blocker Lactisole© is expected to attenuate this effect, as will exendin 9-39 as a competitive GLP-1 antagonist. In addition, we will measure brain activity after fructose administration in order to compare findings to activity after glucose ingestion.

Hypotheses

1) In morbidly obese patients glucose is less potent to trigger the gut-brain axis than in healthy normal-weight population.
2) Intragastric administration of glucose and fructose has different effects on brain activity; fructose must have an alternative way of leading to satiety.

Methods

Study design

fMR after fructose and glucose-stimulation in morbidly obese patients:

The study will be conducted as a placebo-controlled trial with each subject studied on 3 occasions. The test trials will be identical in design except for the intragastral perfusions (glucose 75g, fructose 25g or placebo). Fructose and glucose will be dissolved in 300ml tap water. The order will be balanced and pseudo-random. Each subject will participate in three 1.5 hour study sessions.

Study duration and study site

The study will preferably start in October, 2013. The study duration is then depending on MR-capacity.
Between the sessions patients will have 3-5 days off. Study site: Phase 1 Research Unit, Division of Division of Gastroenterology & Hepatology, University Hospital of Basel, Switzerland.

Recruitment and Screening

Twelve morbidly obese subjects will be recruited. Drop-outs will be replaced. The screening procedure will include the following assessments: a medical interview and a full physical examination. Anthropometric measurements including weight, height, BMI, as well as heart rate and blood pressure will be recorded for all participants.

Inclusion criteria

- 12 males and females
- Body-mass index of ≥ 35
- Age 18-45 years

Exclusion criteria:

- Smoking
- Substance abuse
- Regular intake of medications (except for oral contraceptives)
- Medical or psychiatric illness, especially: diabetes, pace-maker, claustrophobia
- History of gastrointestinal disorders, history of abdominal surgery
- Food allergies, fructose intolerance
- Body piercings that cannot be removed

Study protocol

Overall design

Subjects take part in a 30 min screening session, three ~1.5 h test sessions, and a 30 min end of study (EOS) visit. During the test sessions, subjects will receive an intragastral administration of glucose, fructose or tap water (negative control). The test sessions will be identical in design except for the perfusions. The study will be performed as a randomized, double-blind, placebo-controlled, crossover study, with each subject studied on 3 occasions at least three to five days apart.

Experimental procedure

Each of the test sessions will last ~1.5 h starting at 8:00 AM resp. 9:00 AM. On each experimental session, subjects will be admitted to the Phase 1 Research Unit of the University Hospital Basel in the morning after an overnight fast and will swallow a feeding tube (external diameter 8 french). The tube will be inserted through one of the nostrils into the stomach using a guide wire. The guide wire will be removed and the tube will be firmly attached to the skin behind the ear to prevent further progression of the tube during the treatment. An intravenous catheter will be placed into one forearm. Blood will be drawn for plasma hormone measurements from at 15 or 30 minutes intervals until the end of the study (t = 60 min). Subjects will receive in random order intragastral glucose, fructose or tap water. After administration of the test solution, the feeding tube will immediately be removed and the patient will be placed on the MRI-table. A 3.0 Tesla scanner at the University Hospital of Basel will be used to acquire the neuroimaging data. First, a structural scan will be collected during 6 minutes. Then, functional MR images demonstrating brain activity will be acquired using single shot gradient echo EPI sequence with an echo time of 40ms. Image acquisition will continue for 1 hour after test solution administration. In total, four blood samples will be taken: 2 before administration of test solution, one blood sample after 15 minutes and a last blood sample at 60 min. At each time point, 10 ml blood will be taken into tubes containing EDTA (6 µmol/l), aprotinin (500 kIU/l) and a DPP-IV inhibitor. After centrifugation (3000 rpm, 10 min, 4 °C), plasma samples will be kept frozen at -20°C until analysis. Glucose, insulin, GIP, GLP-1, PYY and ghrelin will be measured. The total blood volume taken during one test day will be 40 ml.

Data Analysis, sample size estimation, statistical analysis of outcomes

The purpose of this study is to gain basic information on brain activity after administration of different carbohydrate solutions and the physiologic regulation of hormone release. Since there is no information available of effect size, sample size of this study was chosen on the basis of practical considerations rather than statistical estimation. However, according to our previous experience with intragastral stimulation of gut hormone release and brain activation, a sample size of 12 subjects will most likely allow to detect large differences between the treatment groups. Descriptive statistics will be used for demographic variables such as age, weight, height, and BMI. Individual hormone concentrations versus time data will be used to obtain GLP-1, PYY, insulin and ghrelin metrics, including maximum/minimum plasma concentrations (Cmax/Cmin), the time of maximal/minimal peptide occurrence (Tmax/Tmin) and the area under the plasma concentration-time profile (AUC). Pharmacokinetic parameters (Cmax, tmax, AUC, etc.) will be obtained using PK Functions for Excel®. AUC values will be calculated by the trapezoidal rule. Differences between treatments will be assessed using either the non-parametric Friedman-test (in case of significant differences followed by pairwise comparison using the Wilcoxon signed ranks test and Bonferroni‘s correction to account for multiple of

comparisons); or the General linear model procedure of repeated-measures ANOVA using simple contrast and Bonferroni correction of P values for multiplicity of comparison. VAS will be analysed by calculating AUC and return to baseline values (interception with y-axis using linear interpolation). Differences will be assessed using the non-parametric Friedman-test or the General linear model procedure of repeated-measures ANOVA. Transformations will be performed before analysis, if response variables are non-normally distributed. All statistical analysis will be done using SPSS for windows software (version 19.0).

Risks and benefits

Participants do not profit directly from this study. MRI is a non-invasive method, with no exposure to radiation.

Compensation

Subjects will receive a financial compensation of 150 CHF for each study day (for the completed trial 450 CHF). Pro rata payments will be done for early discontinuation.

Experimental Design

**Treatments**				
intragastral	**A**	i.g. glucose 75g		
300ml	**B**	i.g. fructose 25g		
	**C**	i.g. tap water		
**Patients**	12 morbidly obese patients			
**Study**	fMRI brain, blood samples for hormone assays,			
	Total: 36 examinations (12x3)			

References

1. Kelly T, Yang W, Chen CS, Reynolds K, He J. Global burden of obesity in 2005 and projections to 2030. *International journal of obesity* 2008; **32**(9): 1431-7.
2. Stein CJ, Colditz GA. The epidemic of obesity. *J Clin Endocrinol Metab* 2004; **89**(6): 2522-5.
3. Sjostrom L, Lindroos AK, Peltonen M, et al. Lifestyle, diabetes, and cardiovascular risk factors 10 years after bariatric surgery. *The New England journal of medicine* 2004; **351**(26): 2683-93.
4. Sjostrom L, Narbro K, Sjostrom CD, et al. Effects of bariatric surgery on mortality in Swedish obese subjects. *The New England journal of medicine* 2007; **357**(8): 741-52.
5. Adams TD, Gress RE, Smith SC, et al. Long-term mortality after gastric bypass surgery. *The New England journal of medicine* 2007; **357**(8): 753-61.
6. Peterli R, Wolnerhanssen B, Peters T, et al. Improvement in glucose metabolism after bariatric surgery: comparison of laparoscopic Roux-en-Y gastric bypass and laparoscopic sleeve gastrectomy: a prospective randomized trial. *Ann Surg* 2009; **250**(2): 234-41.
7. Schmitz O. The GLP-1 concept in the treatment of type 2 diabetes--still standing at the gate of dawn? *J Clin Endocrinol Metab* 2008; **93**(2): 375-7.
8. Batterham RL, Cohen MA, Ellis SM, et al. Inhibition of food intake in obese subjects by peptide YY3-36. *The New England journal of medicine* 2003; **349**(10): 941-8.
9. Beglinger C, Degen L. Gastrointestinal satiety signals in humans--physiologic roles for GLP-1 and PYY? *Physiology & behavior* 2006; **89**(4): 460-4.
10. Gutzwiller JP, Drewe J, Goke B, et al. Glucagon-like peptide-1 promotes satiety and reduces food intake in patients with diabetes mellitus type 2. *The American journal of physiology* 1999; **276**(5 Pt 2): R1541-4.
11. Gutzwiller JP, Goke B, Drewe J, et al. Glucagon-like peptide-1: a potent regulator of food intake in humans. *Gut* 1999; **44**(1): 81-6.
12. Peterli R, Steinert RE, Woelnerhanssen B, et al. Metabolic and Hormonal Changes After Laparoscopic Roux-en-Y Gastric Bypass and Sleeve Gastrectomy: a Randomized, Prospective Trial. *Obes Surg* 2012; **22**(5): 740-8.
13. Chaudhri OB, Parkinson JR, Kuo YT, et al. Differential hypothalamic neuronal activation following peripheral injection of GLP-1 and oxyntomodulin in mice detected by manganese-enhanced magnetic resonance imaging. *Biochemical and biophysical research communications* 2006; **350**(2): 298-306.
14. Parkinson JR, Chaudhri OB, Kuo YT, et al. Differential patterns of neuronal activation in the brainstem and hypothalamus following peripheral injection of GLP-1, oxyntomodulin and lithium chloride in mice detected by manganese-enhanced magnetic resonance imaging (MEMRI). *NeuroImage* 2009; **44**(3): 1022-31.
15. De Silva A, Salem V, Long CJ, et al. The gut hormones PYY 3-36 and GLP-1 7-36 amide reduce food intake and modulate brain activity in appetite centers in humans. *Cell metabolism* 2011; **14**(5): 700-6.
16. Steinert RE, Frey F, Topfer A, Drewe J, Beglinger C. Effects of carbohydrate sugars and artificial sweeteners on appetite and the secretion of gastrointestinal satiety peptides. *The British journal of nutrition* 2011; **105**(9): 1320-8.
17. Sternini C, Anselmi L, Rozengurt E. Enteroendocrine cells: a site of 'taste' in gastrointestinal chemosensing. *Current opinion in endocrinology, diabetes, and obesity* 2008; **15**(1): 73-8.
18. Rozengurt E, Sternini C. Taste receptor signaling in the mammalian gut. *Current opinion in pharmacology* 2007; **7**(6): 557-62.
19. Raybould HE. Gut chemosensing: interactions between gut endocrine cells and visceral afferents. *Autonomic neuroscience : basic & clinical* 2010; **153**(1-2): 41-6.
20. Dockray GJ. Luminal sensing in the gut: an overview. *Journal of physiology and pharmacology : an official journal of the Polish Physiological Society* 2003; **54 Suppl 4**: 9-17.

21. Fujita T. Concept of paraneurons. *Archivum histologicum Japonicum = Nihon soshikigaku kiroku* 1977; **40 Suppl**: 1-12.
22. Newson B, Ahlman H, Dahlstrom A, Nyhus LM. Ultrastructural observations in the rat ileal mucosa of possible epithelial "taste cells" and submucosal sensory neurons. *Acta physiologica Scandinavica* 1982; **114**(2): 161-4.
23. Hofer D, Puschel B, Drenckhahn D. Taste receptor-like cells in the rat gut identified by expression of alpha-gustducin. *Proc Natl Acad Sci U S A* 1996; **93**(13): 6631-4.
24. Dyer J, Salmon KS, Zibrik L, Shirazi-Beechey SP. Expression of sweet taste receptors of the T1R family in the intestinal tract and enteroendocrine cells. *Biochemical Society transactions* 2005; **33**(Pt 1): 302-5.
25. Wu SV, Rozengurt N, Yang M, Young SH, Sinnett-Smith J, Rozengurt E. Expression of bitter taste receptors of the T2R family in the gastrointestinal tract and enteroendocrine STC-1 cells. *Proc Natl Acad Sci U S A* 2002; **99**(4): 2392-7.
26. Rozengurt N, Wu SV, Chen MC, Huang C, Sternini C, Rozengurt E. Colocalization of the alpha-subunit of gustducin with PYY and GLP-1 in L cells of human colon. *American journal of physiology Gastrointestinal and liver physiology* 2006; **291**(5): G792-802.
27. Kokrashvili Z, Mosinger B, Margolskee RF. Taste signaling elements expressed in gut enteroendocrine cells regulate nutrient-responsive secretion of gut hormones. *The American journal of clinical nutrition* 2009; **90**(3): 822S-5S.
28. Jang HJ, Kokrashvili Z, Theodorakis MJ, et al. Gut-expressed gustducin and taste receptors regulate secretion of glucagon-like peptide-1. *Proc Natl Acad Sci U S A* 2007; **104**(38): 15069-74.
29. Margolskee RF, Dyer J, Kokrashvili Z, et al. T1R3 and gustducin in gut sense sugars to regulate expression of Na+-glucose cotransporter 1. *Proc Natl Acad Sci U S A* 2007; **104**(38): 15075-80.
30. Steinert RE, Gerspach AC, Gutmann H, Asarian L, Drewe J, Beglinger C. The functional involvement of gut-expressed sweet taste receptors in glucose-stimulated secretion of glucagon-like peptide-1 (GLP-1) and peptide YY (PYY). *Clinical nutrition* 2011; **30**(4): 524-32.
31. Parks EJ, Skokan LE, Timlin MT, Dingfelder CS. Dietary sugars stimulate fatty acid synthesis in adults. *The Journal of nutrition* 2008; **138**(6): 1039-46.
32. Malik VS, Hu FB. Sweeteners and Risk of Obesity and Type 2 Diabetes: The Role of Sugar-Sweetened Beverages. *Curr Diab Rep* 2012.
33. Hajnal A, Kovacs P, Ahmed T, Meirelles K, Lynch CJ, Cooney RN. Gastric bypass surgery alters behavioral and neural taste functions for sweet taste in obese rats. *American journal of physiology Gastrointestinal and liver physiology* 2010; **299**(4): G967-79.
