# Supplementary material for: Dissociable Behavioral, Physiological and Neural Effects of Acute Glucose and Fructose Ingestion: A Pilot Study
Source: PLoS One. 2015 Jun 24;10(6):e0130280. doi: 10.1371/journal.pone.0130280 (PMC4481317; doi:10.1371/journal.pone.0130280)
Supplement: S1 Table — (PDF) [file pone.0130280.s002.pdf]

Table S1: Treatment differences of resting state functional connectivity to the basal ganglia network relative to placebo

**Treatment comparison**	**Voxel number**	**[1]MNI coordinates (x,y,z)**	**Region**
glucose > placebo	45	(14, 22, 8)	Right caudatus
		(-14, 6, 0)	Left pallidum
		(38, 38, -8)	Orbitofrontal cortex
placebo > glucose	21	(-58, -54, 24)	Angular gyrus
		(-50, -74, 24)	Lateral occipital cortex
		(-14, -70, 40)	Precuneus
fructose > placebo	28	(38, 38, -12)	Orbitofrontal cortex
		(-30, -46, -32)	Cerebellum
		(-38, -62, 0)	Lateral occipital cortex
placebo > fructose	25	(-26, -42, 44)	Left superior parietal lobule
			Planum temporale,
			Supramarginal gyrus
		(38, 30, 8)	Inferior frontal gyrus
		(6, 26, 36)	Paracingulate gyrus

[1]MNI, Montreal Neurological Institute
