# Supplementary figures and images for: Dissociable Behavioral, Physiological and Neural Effects of Acute Glucose and Fructose Ingestion: A Pilot Study
Source: PLoS One. 2015 Jun 24;10(6):e0130280. doi: 10.1371/journal.pone.0130280 (PMC4481317; doi:10.1371/journal.pone.0130280)

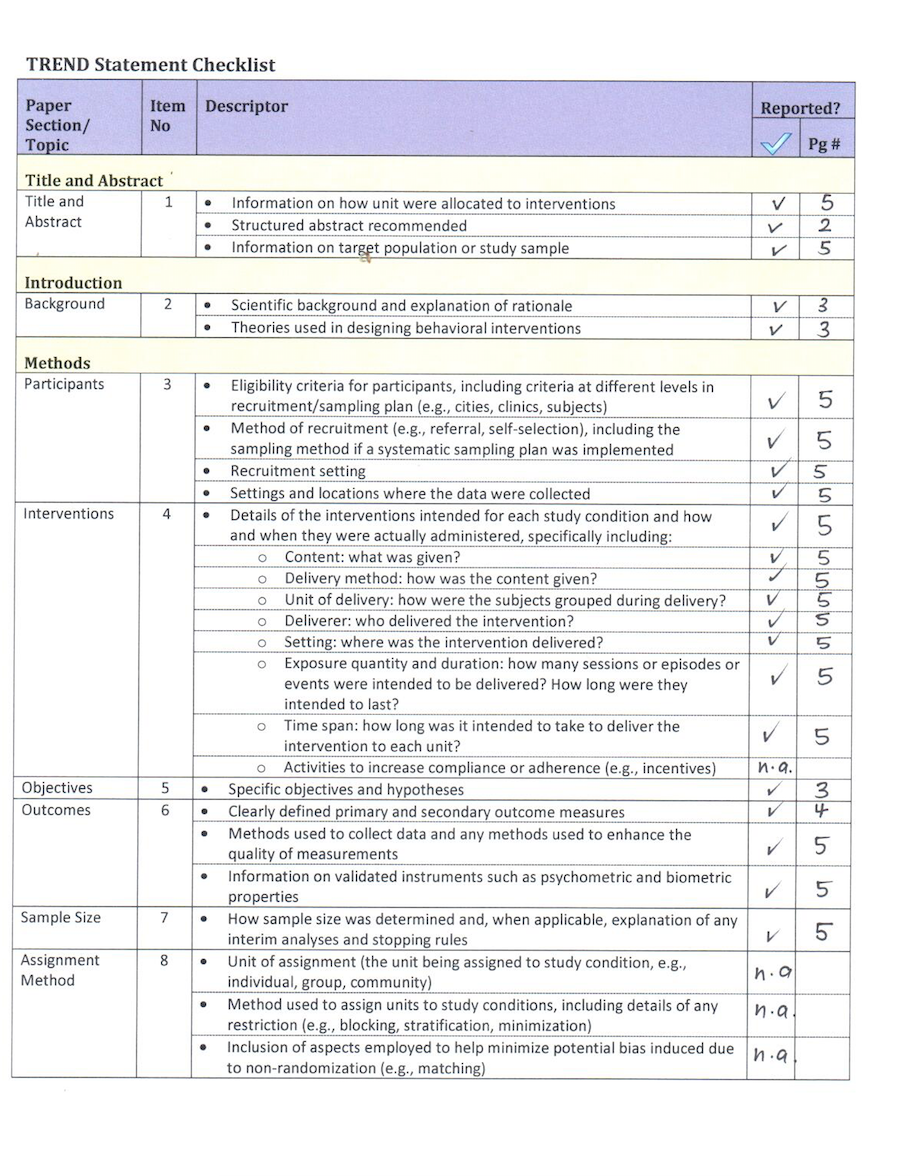

Supplement: S1 Text — (TIFF) [file pone.0130280.s003.tiff]

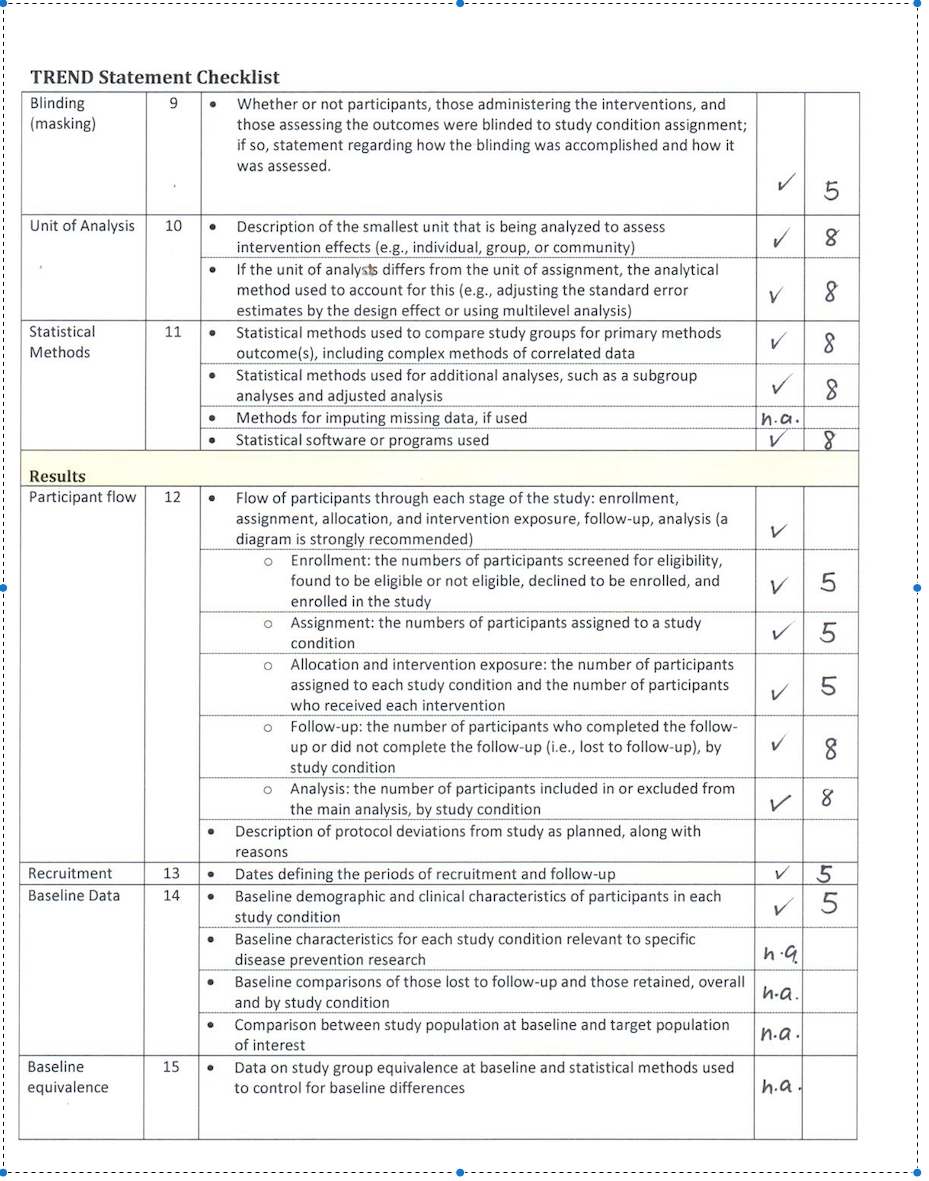

Supplement: S2 Text — (TIFF) [file pone.0130280.s004.tiff]

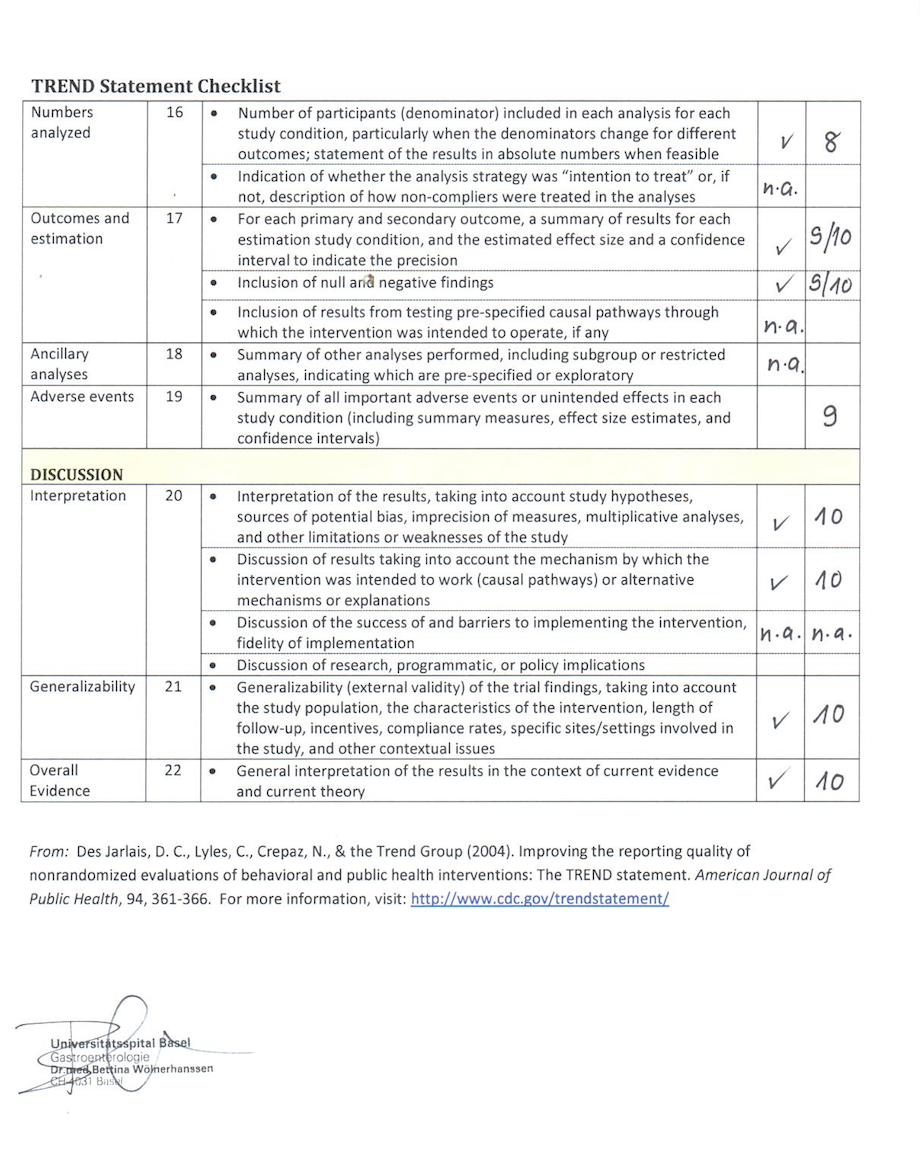

Supplement: S3 Text — (TIFF) [file pone.0130280.s005.tiff]
